# A clustered pulmonary Tuberculosis outbreak at a technical school in Shenzhen, China

**DOI:** 10.3389/fped.2026.1817016

**Published:** 2026-05-07

**Authors:** Jing Tang, Mingbin Xie, Congyang Li, Fan Huang, Liai Peng, Yali Qu, Jinzhou Mei, Zhenyang Liu, Eryong Liu, Yanfang Guo, Yunxia Wang

**Affiliations:** 1 Department of Tuberculosis Control and Prevention, Bao'an District Hospital for Chronic Diseases Prevention and Cure, Shenzhen, Guangdong Province, China; 2 School of Public Health (Shenzhen), Sun Yat-sen University, Shenzhen, Guangdong Province, China; 3 National Center for TB Control and Prevention, Chinese Center for Disease Control and Prevention, Beijing, China

**Keywords:** school tuberculosis outbreak, contact investigation, syndromic surveillance, TB, WGS

## Abstract

**Background:**

On 11 June 2025, a clustered outbreak of pulmonary tuberculosis (TB) was detected in a vocational school class in Shenzhen.

**Methods:**

A two-phase screening investigation was conducted in accordance with Chinese tuberculosis prevention and treatment guidelines. Screening procedures included symptom assessment, Interferon-Gamma Release Assays (IGRA), and chest radiography (CXR). Confirmed, clinically diagnosed, and suspected cases were identified by specialized pulmonologists following the Diagnostic Criteria for Pulmonary Tuberculosis (WS 288–2017). To assess transmission patterns, a comparative analysis of dormitory and class distributions among individuals with a positive IGRA result was performed. Additionally, whole genome sequencing (WGS) of clinical isolates was conducted to determine genetic relatedness and infer potential transmission pathways.

**Results:**

After the initial report of two cases, two rounds of screening were conducted at the school. In the first round, 453 individuals were screened. IGRA yielded valid results for 421 individuals, with a positivity rate of 9.3% (39/421); in the second round, the IGRA positivity rate was 5.1% (9/176). The abnormal CXR rates were 2.4% (11/453) and 1.1% (2/176), respectively. Across both screenings, a total of 7 cases of active tuberculosis were confirmed (including one negative for IGRA), and 42 cases of latent infection were identified. A comparative analysis of the dormitory and class distributions among individuals with a positive IGRA result showed that the IGRA positivity rate in the affected class was 62.2 % (23/37), significantly exceeding rates observed in other classes (P < 0.05). The four dormitories with the highest positivity rates were all occupied by students from Class 2302 (Room 310: 100%, Room 309: 62.5%, Room 506: 50.0%, and Room 308: 37.5%). WGS analysis revealed that five isolates belonged to the Beijing genotype and exhibited high genetic homology with minimal divergence, confirming a single clonal outbreak with slight genetic variation.

**Conclusions:**

This outbreak stemmed from intertwined factors: close contact, poor ventilation, delayed detection, and insufficient prevention. Early syndromic surveillance, enhanced health education for students and staff, improved environmental hygiene, and timely implementation of preventive treatment for latent tuberculosis infection are vital strategies for preventing and controlling TB clusters in educational settings.

## Introduction

The Global Tuberculosis Report 2025 highlights that tuberculosis (TB) remains a significant global public health concern, causing around 1.23 million deaths in 2024, which is twice the mortality rate associated with HIV/AIDS (1). Among the 10.7 million new TB cases reported in 2024, children and adolescents under the age of 15 years old constituted 11%, while this demographic accounted for approximately 172,000 TB-related deaths, representing 16% of the total fatalities (1). Adolescents are especially susceptible to *Mycobacterium tuberculosis* transmission due to physiological changes such as hormonal fluctuations, alterations in social behavior, immune system variability, and the densely populated settings typical of school environments (2–6). As one of the 30 high-burden TB countries, China has emphasized the implementation of school-based TB prevention strategies; nonetheless, the occurrence of clustered outbreaks continues to pose a substantial public health challenge.

TB outbreaks are notably prevalent in environments characterized by high population density and frequent close interpersonal contact. Educational institutions, particularly schools, which are typified by crowded conditions and intensive social interactions, represent high-risk settings for TB transmission (5, 7). During a 2016 outbreak at a high school in Shaoxing, Zhejiang Province, a diagnostic delay of three months in the index case resulted in 52 related cases, encompassing suspected, confirmed, and latent infections. Students sharing the same classroom or dormitory exhibited significantly increased risk, and some individuals with latent infections progressed to active TB after declining preventive treatment (8). Similarly, a 2018 outbreak in Shenzhen was precipitated by a misdiagnosis of the index case, leading to five additional confirmed cases within the same and adjacent classes, inadequate ventilation, and deficiencies in control measures further exacerbated transmission (9). Diagnostic delays also contributed to cluster formation in a boarding high school in Jiangsu in 2018 (10). More recently, in 2024, the absence of routine health monitoring at a boarding high school in Guigang, Guangxi, culminated in an outbreak of isoniazid-resistant TB affecting multiple grades, with evidence indicating transmission from households to the school setting (11). Moreover, numerous domestic and international studies have documented TB clustering across diverse educational contexts (6, 12–15). Collectively, the extant literature suggests that school-based TB outbreaks predominantly occur within close-contact units, such as classrooms and dormitories, where crowded conditions and proximity constitute critical environmental facilitators. Principal risk factors include close-contact exposure, insufficient ventilation, and diagnostic delays in index cases. Accordingly, the standardized management of latent TB infection is essential for interrupting chains. Nonetheless, current prevention and control strategies tailored to these specific settings warrant further refinement and enhancement.

This research focused on a clustered outbreak of pulmonary tuberculosis that transpired in June 2025 at a boarding vocational school in Shenzhen. Using a retrospective analysis of data from two rounds of screening, which identified four laboratory-confirmed and five clinically diagnosed cases, the study sought to investigate the epidemiological features and contributing factors of this clustered infection. The primary aim is to inform strategic approaches for the early detection and targeted management of tuberculosis among adolescent populations within educational institutions and comparable specialized environments in China.

## Materials and methods

### Case definition and contact classification

TB cases were classified into three categories: confirmed, clinically diagnosed, and suspected cases. All diagnostic assessments and classifications were conducted by specialized pulmonologists in accordance with the *Guidelines for the Prevention and Treatment of Tuberculosis in China*, the *Diagnosis for Pulmonary Tuberculosis* (WS 288–2017) (16), and the *Classification of Tuberculosis* (WS 196–2017) (17).

1. Confirmed cases were identified based on bacteriological evidence, such as positive sputum smear microscopy, culture, or molecular diagnostic tests, or pathological evidence.
2. Clinically diagnosed cases were characterized by the absence of bacteriological confirmation but presented with typical clinical manifestations and radiological features consistent with TB, supported by auxiliary tests (e.g., positive Purified Protein Derivative (PPD)/ Interferon-Gamma Release Assays (IGRA)), after excluding other respiratory conditions.
3. Suspected cases included individuals exhibiting symptoms or radiological findings suggestive of TB, warranting further diagnostic investigation.

The initial active pulmonary TB case identified in this school outbreak was designated as the index case. Contacts were categorized into three groups based on the duration, intensity, and nature of their exposure to the index case (18):

1. Close contacts: Teachers and students sharing the same classroom or dormitory with the index case, as well as individuals with close social interactions.
2. General contacts: Persons occupying the same teaching or dormitory floor for academic or residential purposes.
3. Occasional contacts: All other students and staff members who did not fulfill the criteria for close or general contact.

### Epidemiological investigation and screening protocols

After the detection of the initial two cases, a comprehensive field epidemiological investigation and contact screening were implemented in accordance with the 2020 edition of the Guidelines for Tuberculosis Control in Chinese Schools (2020 Edition) (18). The investigation comprised the following four key stages:

1. Contact Identification. The contact population was delineated and stratified into three categories: (1) Close Contacts: classmates, students residing in adjacent dormitories, and social acquaintances; (2) General C contacts: students occupying the same teaching or dormitory floors and members of extracurricular clubs); (3) Occasional Contacts: other students, staff members, and canteen personnel.
2. Screening Components and Methodological Approach. Screening procedures for close contact encompassed symptom assessment, IGRA or PPD, and chest radiography (CXR). Subjects requiring further differential diagnosis underwent additional evaluation through Computed Tomography (CT) scans. For individuals presenting with symptoms suggestive of TB, a positive IGRA or strong PPD-positive result, or abnormal CXR findings, three sputum specimens were collected and subjected to both microscopic examination and culture. It should be noted that in this study, only IGRA was used for screening of reported cases, whereas PPD was primarily used for Case0 associated with the reported cases. In actual screening practice, either method is acceptable, and only one needs to be selected. The choice of method may be determined based on practical circumstances (e.g., funding, stock availability).
3. Post-Screening Management. A standardized protocol for clinical management and follow-up was applied to all individuals diagnosed with active tuberculosis, those suspected of infection, and participants with either positive or negative IGRA or PPD results, in accordance with national health guidelines (18).
4. Follow-up Screening of Close Contacts. A subsequent screening was conducted three months following the initial outbreak to detect cases with delayed onset or infections occurring during the window period. This follow-up targeted a cohort of 195 individuals, comprising those who tested IGRA-positive in the initial screening, as well as the original groups of close and general contacts.

### Genomic and phylogenetic analysis

Sputum samples were collected from patients with positive cultures and subjected to whole-genome sequencing. DNA extraction was performed, followed by whole-genome sequencing on the Illumina NovaSeq X Plus platform with paired-end reads, achieving an average sequencing depth of 200×.

#### Quality control

Raw sequencing data were quality-controlled using Fastp (v1.1.0) (19) with parameters: cut_front, cut_tail, minimum length 50 bp, and detect_adapter_for_pe.

### Sequence alignment and variant calling

Cleaned sequences were aligned to the *Mycobacterium tuberculosis* reference genome H37Rv using BWA-MEM (v0.7.19-r1273) (20). Alignments were sorted, duplicate-marked, and indexed using SAMtools (v1.23) (21). Variant calling was performed using the BCFtools (v1.23) (22) mpileup pipeline with parameters: minimum base quality 20, minimum mapping quality 20, max depth 1000, and ploidy set to 1 (haploid). Detected variants were filtered using stringent criteria: a quality score below 50, a read depth less than 20, a mapping quality below 30, fewer than 2 supporting reads on either the forward or reverse strand, or an alternate allele frequency lower than 90%.

### Genotyping and drug resistance profiling

Whole-genome drug resistance profiling and lineage identification were conducted using TB-Profiler (v4.4.2) (23, 24). Analysis was performed using the default parameters.

### Core genome extraction and phylogenetic analysis

Core genome was extracted using strict site selection criteria: single nucleotide polymorphism sites present in at least two samples (excluding indels and recombinant regions). High-quality variant sites were converted to a multiple sequence alignment using BCFtools (22). Pairwise genetic distances between samples were calculated based on the core SNP matrix using SNP-dists (v1.2.0) (https://github.com/tseemann/snp-dists). A phylogenetic tree was constructed and visualized using the R package ggtree (25–27).

### Statistical analysis

All statistical evaluations were conducted using R software (version 4.5.1). Categorical variables were compiled and compared utilizing either the Chi-square test or Fisher's exact test, depending on data suitability, incorporating Bonferroni adjustments for multiple comparisons to maintain an overall significance level of 0.05.

## Results

### General overview of the outbreak

The technical school impacted by the epidemic consisted of 11 classes, employed 47 staff members across teachers, administrative, logistical, and property management roles, and enrolled a total of 416 students. The first two cases in the same class were subsequently diagnosed with secondary pulmonary tuberculosis in June 2025. Screening was promptly initiated. As the epidemic evolved, the scope of the first-round screening was progressively broadened in accordance with national health guidelines (18): Phase I targeted close contacts exclusively, while Phases II and III progressively included general and occasional contacts, respectively. The second round of screening was carried out three months subsequent to the initial screening, primarily focusing on individuals who tested positive for IGRA and close as well as general contacts identified during the first round. Following two stages of screening, a total of nine cases of active pulmonary tuberculosis were identified, comprising four bacteriologically confirmed and five clinically diagnosed instances, and 42 latent infection cases. The affected group included eight males and one female, with eight individuals residing in boarding accommodations and one attending as a day student, and all of all of in the same class (Table 1). Among the 42 latent infected individuals in this case, 32 received preventive treatment, 4 refused preventive treatment, and 6 individuals were ineligible for preventive medication due to contraindications (e.g., abnormal liver function). The latent infected individuals receiving preventive treatment have not reported active pulmonary tuberculosis so far.

**Table 1:** Demographic and clinical characteristics of nine active pulmonary tuberculosis cases.

Case	Sex	Age	Sym	Radiographic findings	IGRA	Sputum Smear	Molecular	Culture
Case1	M	17	Non	Patchy, spotty, and linear strand-like opacities are seen in the upper and middle lung zones bilaterally.	Pos	Neg	Neg	Pos
Case2	M	17	Non	A faint, patchy opacity is suspected at the left lung apex.	Pos	Neg	Neg	Neg
Case3	M	16	Cough	Multiple patchy, nodular, and linear opacities are seen in both lungs; cavitation is noted within some of the lesions.	Pos	Neg	Pos	Pos
Case4	M	17	Non	An irregular patchy and linear opacity with heterogeneous density in the apical-posterior segment of the right upper lobe.	Pos	Neg	Pos	Pos
Case5	M	17	Non	Blunting of the left costophrenic angle.	Neg	Neg	Neg	Neg
Case6	M	17	Cough, fever	Blunting of the left costophrenic angle.	Pos	Neg	Pos	Pos
Case7	M	18	Non	Blunting of the right costophrenic angle.	Pos	Neg	Neg	Neg
Case8	M	17	Non	Scattered, patchy, ill-defined opacities with increased density are seen in the subpleural region of the anterior segment of the left upper lobe; a small left pleural effusion is present.	Pos	Neg	Neg	Neg
Case9	F	17	Non	A few patchy, linear, and nodular opacities with slightly increased density in the apical segment of the right upper lobe, the lateral basal segment of the right lower lobe, and other scattered areas	Pos	Neg	Neg	Neg

Tuberculosis cases: Sym, symptoms; Pos, positive, Neg, negative.

### Initial epidemiological investigation and screening

In June 2025, following the diagnosis of two secondary pulmonary tuberculosis cases in the same class, the initial screening phase was initiated. Based on epidemic progression, the screening was conducted in a phased manner: beginning with close contacts (*n* = 61), which included classmates, students residing in adjacent dormitories, and social acquaintances. This was subsequently extended to general contacts (*n* = 165), encompassing students on the same teaching or dormitory floors as well as members of extracurricular clubs. Ultimately, the screening encompassed occasional contacts (*n* = 227), comprising other students, staff members, and canteen personnel, thereby ensuring coverage of the entire population of teachers and students within the school. Ten students did not participate in the screening due to reasons such as leave or internship. All individuals screened were aged 15 years or older. The initial screening phase should include 463 participants for TB evaluation, but ultimately enrolled 453, comprising 406 students (mean age 16.7 ± 0.8 years; 57.6% male) and 47 staff members (mean age 39.0 ± 12.3 years; 46.8% male) (Supplementary Table 1). During this phase, the completion rates were recorded as 97.6% (Supplementary Table 2). But there are 32 cases with no valid reported IGRA results (28 students and 4 staff). Among them, 34 students (34/378 = 9.0%) and 5 faculty members (5/43 = 11.6%) tested positive for IGRA. All individuals underwent chest x-ray examination. The abnormal CXR rates were 2.40% (11/453). Sputum tests were conducted on individuals who tested positive for IGRA or had an abnormal chest radiograph. A total of 44 people participated in the sputum tests. Among them, 0 tested positive for sputum smear, 3 for molecular testing and sputum culture. In the first round of screening, 6 cases of tuberculosis were finally confirmed.

### Secondary screening

Between late August and early September 2025, approximately three months following the initial outbreak, a second phase of screening was implemented. Individuals who tested positive for IGRA during the initial screening underwent chest CT scans. Concurrently, close and general contacts who were initially IGRA-negative were subjected to repeat IGRA testing. Out of 195 individuals scheduled for IGRA re-testing, 176 completed the procedure, yielding 9 positives (9/176; the remaining 19 individuals were unavailable due to participation in internships or withdrawal from the institution. Excluding previously confirmed cases, a total of 43 individuals—34 from the first screening round (excluding 5 confirmed cases) and 9 from the second—were identified for follow-up chest CT examinations. CT scans identified two cases exhibiting tuberculosis-related radiographic abnormalities, one of which was classified as pulmonary TB, while the other was subsequently excluded.

### Analysis of screening results

Combining data from both screening rounds, the overall IGRA positivity rate within the school population was 11.4% (48 out of 421), and the CXR Abnormalities rate was 2.9% (13 out of 453) (Supplementary Table 3). When stratified by role, the IGRA positivity rate was 10.6% (40/378) among students and 18.6% (8/43) among staff members. No statistically significant difference was found between students and staff (*OR*=0.52, *P* = 0.128, 95% *CI* = (0.22, 1.39); Table 2), whereas the difference between staff and Class 2302, the outbreak-affected class, was statistically significant (X^2^ = 14.12, *P* < 0.01 *OR* = 0.14,95% *CI* = (0.04, 0.42); Table 2). Similarly, the rates of CXR Abnormalities were comparable, at 3.0% (12/406) among students and 2.1% (1/47) among staff.

**Table 2:** Comparison of screening results (IGRA and CXR) between students, staff, and class 2302.

Group	IGRA Positives/Number Tested (Positivity Rate)	CXR Abnormalities/Number Tested (Abnormality Rate)
Students	40/378 (10.6%)	12/406 (3.0%)
Staff	8/43 (18.6%)	1/47 (2.1%)
Class 2302 (Students)	23/37 (62.2%)	8/37 (21.6%)

Staff vs. Class 2302: Chi-square test, X^2^ = 14.12, *P* < 0.01 OR = 0.14,95% CI = (0.04, 0.42).

Students vs. Staff: Fisher’s exact test, *OR*=0.52, *P* = 0.128, 95% CI = (0.22, 1.39).

Subsequent analyses revealed distinct patterns in the sex-based distribution of IGRA positivity across different subgroups. Among students, the IGRA positivity rate was higher in males (13.2%, 29/220) compared to females (7.0%, 11/158); however, this difference did not achieve statistical significance (*χ²* = 3.13, *P* = 0.077, *OR* = 0.49,95% *CI* = (0.22, 1.06), Table 3). Similarly, within the staff cohort, males exhibited a greater positivity rate (28.6%, 6/21) relative to females (9.1%, 2/22), though this difference was also not statistically significant (Fisher's exact test, OR=0.26, *P* = 0.132, 95% CI = (0.02, 1.71); see Table 3).

**Table 3:** Distribution of screening results (IGRA and CXR) stratified by sex Among students and staff.

Group	Gender	IGRA Positives/tested(%)	CXR Abnormal/tested(%)
Student	Female	11/158 (7.0%)	5/172 (2.9%)
	Male	29/220 (13.2%)	7/234 (3.0%)
Staff	Female	2/22 (9.1%)	1/25 (4.0%)
	Male	6/21 (28.6%)	0/22 (0.0%)

For student sex differences: Chi-square test, X^2^= 3.13, *P* = 0.077, OR = 0.49,95% CI = (0.22, 1.06); for staff sex differences: Fisher’s exact test, *OR*=0.26, *P* = 0.132, 95% CI = (0.02, 1.71).

Analysis at the class level revealed a markedly uneven distribution of IGRA positivity. In particular, Class 2302 displayed an exceptionally elevated IGRA positivity rate of 62.2% (23/37), alongside a CXR Abnormal rate of 16.2% (6/37). Chi-square tests with Bonferroni adjustments for multiple comparisons indicated that the IGRA positivity rate in Class 2302 was significantly greater than those observed in Class 2301 (12.5%, adjusted *P* < 0.001), Class 2401 (9.1%, adjusted *P* < 0.001), Class 2304 (7.1%, adjusted *P* = 0.012), as well as several other classes exhibiting 0% positivity (adjusted *P* < 0.001; refer to Table 4).

**Table 4:** Comparative analysis of IGRA positivity rates among major classes (students only).

Class	IGRA Positives/Number Tested (Positivity Rate)
2302	23/37 (62.2%)
2301	6/48 (12.5%)***
2401	4/44 (9.1%)***
2304	1/14 (7.1%)*
2305	3/51 (5.9%)***
2403	2/39 (5.1%)***
2407	1/35 (2.9%)**
2303	0/4 (0.0%)
2402	0/43 (0.0%)***
2404	0/53 (0.0%)***

Comparisons were made with Class 2302 using the Chi-square test. Multiple comparisons were adjusted using the Bonferroni correction.

Classes with significant differences: Class 2301 (X^2^ = 20.77, adjusted *P* *<* *0.001*); Class 2401 (X^2^ = 23.14, adjusted *P* *<* *0.001*); Class 2304 (X^2^ = 10.23, adjusted *P* = 0.012).

* *P* *<* *0.05*

** *P* *<* *0.01*

*** *P* *<* *0.001*

A comprehensive examination of the student dormitories revealed a marked spatial clustering of tuberculosis (TB) infection. The infection was concentrated in particular clusters: notably, the four dormitories with the highest positivity rates were all inhabited by students from Class 2302. Specifically, Room 310 in Juxian Building exhibited a 100.0% IGRA positivity rate (5/5), alongside a 40.0% incidence of CXR Abnormal 3/5. Furthermore, Rooms 309 and 506 showed positive rates of 62.5% and 50.0%, while Room 308 had a rate of 37.5% (3/8) (Supplementary Table 4). The comparison results between other non-2302 class dormitories and the main epidemic-related dormitory 310 show that, although the *P* value is not significant after multiple adjustments (Supplementary Table 5), the spatial clustering trend is already quite evident. Additionally, the presence of chest radiograph abnormalities only in the dormitories related to Class 2302 further underscores its clustering characteristics.

Conversely, several other dormitories reported no positive cases. Although the differences in positivity rates across dormitories did not reach statistical significance after adjustment for multiple comparisons, the data nonetheless indicated a pronounced trend toward concentrated infection intensity in specific rooms, particularly those allocated to Class 2302. This observation aligns with the class-level analysis, suggesting that TB infection not only clusters at the classroom level but also exhibits distinct spatial aggregation patterns within dormitory settings.

### A retrospective investigation

A retrospective investigation identified a student from the same class (designated Case 0) who had been diagnosed with bacteriologically confirmed pulmonary tuberculosis in January 2025. Although Case 0 was on medical leave, they attended a social event in mid-May 2025 alongside several classmates, including Cases 1, 2, and 3. This gathering constituted the principal exposure event precipitating the current outbreak. And this student also shared accommodation in Room 310. In the screening of case 0, a total of 51 close contacts were required to undergo screening. Among them, 45 underwent PPD screening, 4 underwent IGRA screening, and 2 did not receive relevant infection-related testing; all 51 individuals completed CXR screening. The PPD test results showed 15 positive cases, 7 moderately positive cases, and 4 strongly positive cases; 2 cases were positive on the IGRA test. CXR screening revealed non-tuberculous abnormalities in 3 cases. Of the 4 cases of strong PPD positivity, 2 cases of IGRA positivity were advised to take preventive medication, but only one teacher agreed and completed the medication. In the screening four months later (during this epidemic screening), two students with strong PPD-positive results and one student with a positive IGRA result in the screening of Case 0 were diagnosed as cases.

### Epidemiological features and transmission dynamics

**Figure 1:**
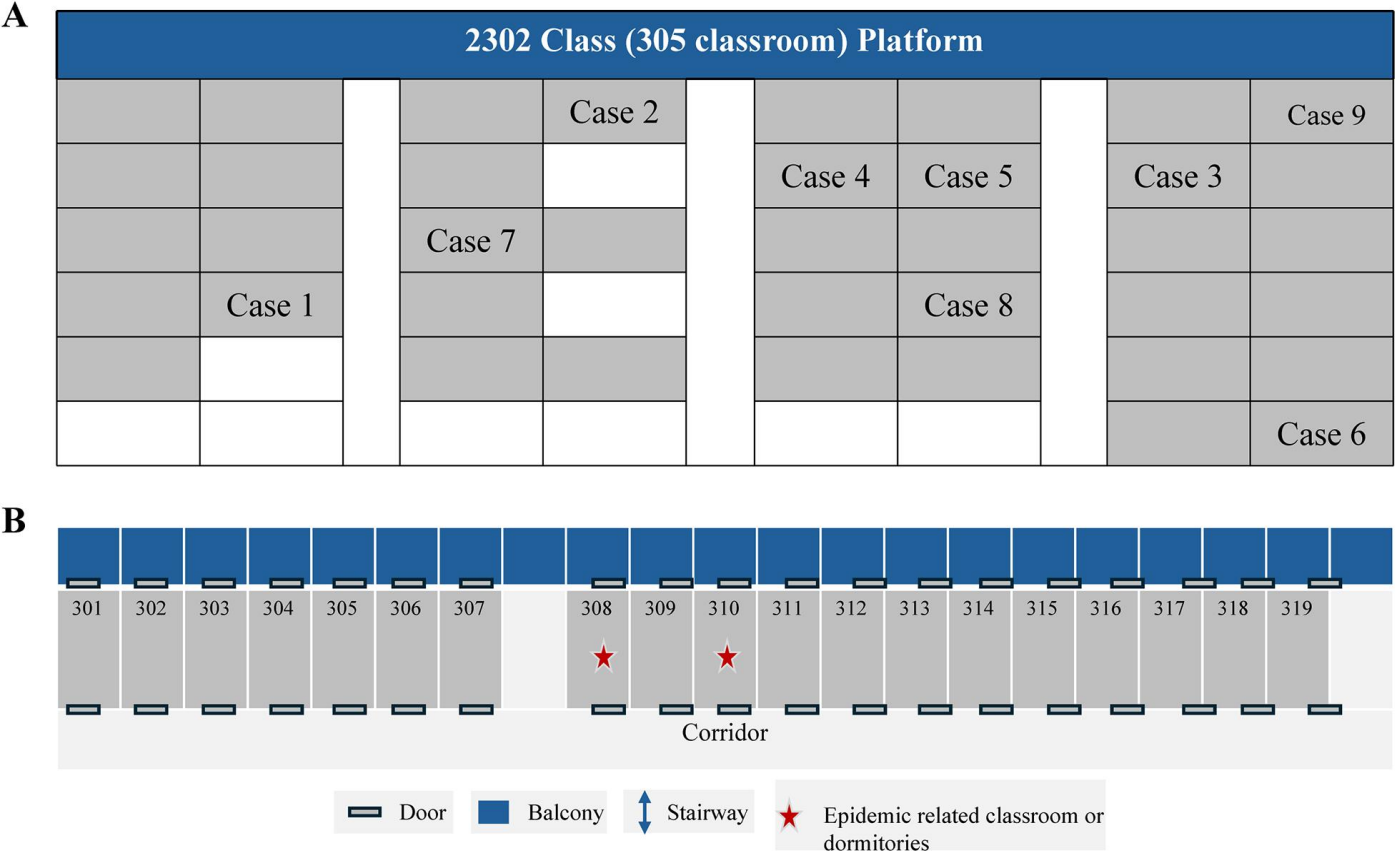
Spatial distribution of TB cases in the classroom **(A)** and dormitory settings **(B)**.

The school is supported by a full-time school health coordinator. The classrooms are located within a repurposed industrial facility, featuring a central corridor with classrooms arranged on both sides. Although cross-ventilation is sufficient when both classroom and laboratory doors are open, the routine practice of keeping windows and doors closed during the summer months to preserve air conditioning has led to suboptimal ventilation conditions. The outbreak was primarily confined to the index case's classroom and dormitory. The spatial distribution of these cases within the classroom and dormitory settings is depicted in Figures 1A, B. Environmental evaluation of the classroom (Room 305) revealed that the space was a converted workshop of approximately 40 square meters, accommodating 39 students. The classroom contained four windows and four UV germicidal lamps installed at the recommended height; however, the lamps were heavily dust-laden and lacked documented routine maintenance or cleaning. The room did not have an exhaust ventilation system (Figure 1A). Although cross-ventilation was feasible when the door to the adjacent machine room was open, windows and doors remained closed during the summer months to preserve air conditioning, even during breaks, resulting in persistently poor air exchange.

Spatial analysis demonstrated that Cases 1, 2, 3, 6, and 8 shared accommodations in Room 310 and resided at the school from Sunday through Thursday. Although Room 310 featured a balcony, wall-mounted air conditioning units, and ceiling fans, it lacked mechanical exhaust ventilation and ultraviolet (UV) disinfection systems; preventive disinfection was conducted only monthly by an external service provider. Students' activities while on campus were largely restricted to the classroom and dormitory, with frequent social interactions occurring between adjacent dormitory rooms (308, 309, and 311) (Figure 1B).

In conclusion, this outbreak was driven by the combined effects of prolonged close contact, insufficient ventilation within residential and educational settings, and inadequate disinfection practices. These findings highlight the critical importance of enhancing early screening protocols, promoting health literacy among students and staff, optimizing campus environmental conditions, and establishing rapid response mechanisms as essential strategies to prevent and control clustered TB outbreaks in educational institutions.

### Phylogenetic analysis

**Figure 2:**
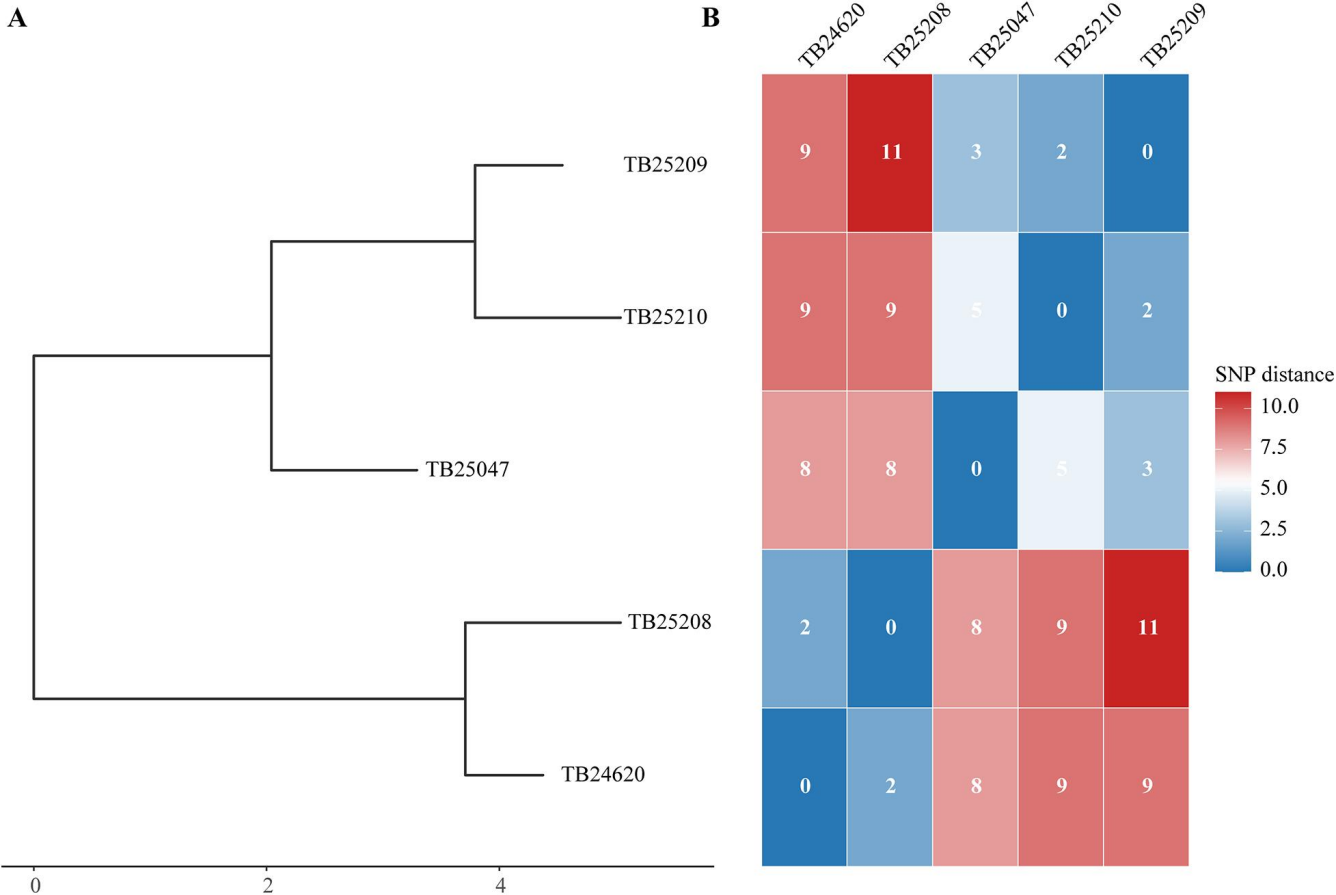
Phylogenetic relationship and pairwise SNP distances of five *Mycobacterium tuberculosis* isolates. **(A)**: Neighbor-joining phylogenetic tree reconstructed from the SNP distance matrix. The tree is midpoint-rooted for clarity; branch lengths represent the cumulative number of SNP substitutions according to the scale bar (top left), and numbers above branches indicate the corresponding SNP distances (rounded to 1 decimal place). **(B)**: Adjacent heatmap showing the pairwise raw SNP distance matrix. Each cell is colour-coded according to the gradient (blue-white-red, with the midpoint set at the median distance) and contains the exact SNP distance value.

Whole-genome sequencing was performed on sputum isolates from five culture-positive patients (Case0, Case1, Case3, Case4, and Case6), corresponding to strains TB24620, TB25209, TB25208, TB25210, and TB25047. The sequencing results revealed that all five cases belonged to the Beijing genotype and were highly homologous. The phylogenetic tree, together with the adjacent heatmap, clearly delineates the micro-evolutionary relationships among the isolates. Among the five strains, pairwise SNP distances of 2 bp were observed for TB24620 vs. TB25208 and TB25209 vs. TB25210, suggesting possible recent common transmission sources for these pairs. In contrast, the largest distance (11 bp) was found between TB25208 and TB25209, indicating a relatively pronounced genetic divergence. The pairwise SNP distances ranged from 2 to 11 bp, with a mean of 6.6 bp and a median of 8 bp (Figure 2). Overall, the five Beijing genotype *Mycobacterium tuberculosis* strains exhibited high consistency at the genomic level, with close phylogenetic relationships, belonging to the same evolutionary lineage (2.2.1). Genetic distance data further supported that they were slightly differentiated strains within the same clonal group, indicating the dynamics of this outbreak.

## Discussion

The management of this tuberculosis outbreak extended over two academic semesters. The initial screening was conducted during the Spring semester of 2025, encompassing the entire school population at that time (*N* = 453, according to the official school registry), which identified eight active pulmonary tuberculosis cases. To identify any late-emerging secondary cases or individuals who may have been within the immunological “window period” of *Mycobacterium tuberculosis* infection during the initial screening, a second round of testing was implemented in the Autumn semester of 2025. This follow-up targeted 176 individuals, including both close and general contacts identified during the first screening, resulting in the detection of one additional case. In total, nine active tuberculosis cases had been documented to date, comprising four bacteriologically confirmed and five clinically diagnosed cases.

The outbreak showed distinct clustering in classrooms and dormitories. All cases were from the same class, with five sharing a dormitory. Case 0, diagnosed in January 2025, carried a strain genetically homologous to other cases, and despite being on medical leave, attended social gatherings such as a birthday party, establishing clear close contact. Daily symptom monitoring was inadequate, and ventilation and disinfection in classrooms and dormitories were suboptimal. During screening for Case 0, two students with strong-positive PPD cases and one student with a positive IGRA result were diagnosed with active tuberculosis four months later. Preventive treatment was recommended to four individuals with strong-positive PPD results and two with positive IGRA results, but only one teacher completed treatment. Students declined due to concerns about side effects or a belief that latent infection does not require treatment; some clinicians also expressed reservations, and certain parents were advised that treatment was unnecessary, further hindering uptake. In summary, this transmission event resulted from multiple interacting factors: prolonged close contact and poor ventilation in shared housing and educational environments created ideal conditions for airborne transmission; inadequate daily symptom monitoring delayed case detection; and the lack of effective disinfection and preventive treatment left the transmission chain unbroken, turning latent infections into ticking time bombs. These four factors were intertwined, driving an ordinary imported case into a clustered outbreak involving multiple individuals.

Comparing the causes of this outbreak with previous school-related tuberculosis outbreaks reported both domestically and internationally revealed a high degree of similarity in core risk factors and common shortcomings in prevention and control. Studies both in China and abroad consistently confirmed that prolonged close contact, poorly ventilated environments, ineffective symptom monitoring, and low coverage of preventive treatment constitute the four pillar causes of school outbreaks (5, 7–9, 28–30). For instance, a study in Wuhan found that “boarding at school” increased the risk of an outbreak (29), while in a South Korean high school outbreak, close contact exceeding 10 h per week raised the infection risk (28). Similarly, analyses of multiple outbreaks in China have identified perfunctory morning inspections and delayed case detection as key contributing factors (29, 31). In addition, low coverage of preventive treatment has been pointed out as a critical factor contributing to sustained transmission in school settings (32). A study covering 136 school tuberculosis outbreaks across six cities in Jiangsu Province from 2019 to 2021 further demonstrated that preventive treatment significantly reduced the incidence of tuberculosis among adolescent close contacts (33). The risk factors of this epidemic are highly similar to those reported in previous outbreaks, but it exposed intertwined systemic deficiencies in four aspects: environmental ventilation deficiencies, ineffective disinfection equipment, inadequate morning/afternoon inspections and absence tracking due to illness, and low adherence to preventive treatment. These shortcomings offered profound lessons for future tuberculosis prevention and control in schools: it is imperative to transform “effective ventilation” from a qualitative requirement into a quantitative standard, shift “disinfection measures” from a configuration-oriented approach to an outcome-oriented one, convert “morning/afternoon inspections and absence tracking due to illness” from mere procedural formality into substantive response, and elevate “preventive treatment” from a medical recommendation to a core target of health education and intervention management.

The WGS analysis results of the five strains in this case indicated that the outbreak was dominated by a single predominant genotype, with the Beijing genotype (Lineage 2) holding an absolute advantage, presenting a typical “monoclonal transmission” pattern, with minimal single nucleotide polymorphism (SNP) differences (≤12 SNPs) among strains within the transmission cluster. Compared with isolates from school outbreaks reported in domestic and international literature, the molecular epidemiological characteristics are highly consistent: among student isolates from a Beijing university between 2004 and 2023, Lineage 2 was the predominant genotype (34); in school outbreaks in Guangzhou from 2015 to 2019, Lineage 2 accounted for 79.26% (35); and a study in Inner Mongolia further specified that sub-lineage 2.2.1 accounted for 89.0% of Lineage 2 (36), which is completely consistent with the subtype of the strains in this case. The predominance of the Beijing genotype is likely driven by its high transmissibility, greater virulence, and higher risk of drug resistance (36, 37). Traditional symptom screening and epidemiological investigation often underestimate the scale of outbreaks and fail to reconstruct transmission networks effectively, whereas WGS technology can precisely reconstruct transmission networks, revealing that the scope of transmission extends far beyond classmates to include school bus occupants, dormitory residents, extracurricular activity participants, and household contacts. In general, the application of advanced technologies such as WGS will open up new avenues for tuberculosis screening, accurate transmission network reconstruction, and optimization of prevention and control strategies.

To effectively prevent and control the transmission of tuberculosis (TB) in campus settings, a comprehensive strategy integrating health monitoring, environmental management, and health education is recommended (18):

First, stringent TB screening must be implemented during entry physical examinations for new students and staff, as well as during routine annual check-ups, to ensure early detection and a proactive defense. Second, the daily morning check-in system, along with the registry for sickness-related absences and subsequent follow-up of underlying causes, should be optimized at the class level. This will strengthen symptomatic monitoring and early warning systems, ensuring timely referral of suspected cases and diligent tracking of diagnostic outcomes. Furthermore, targeted TB prevention education should be conducted during key health promotion periods to enhance health literacy and self-protection capabilities among students and staff, thereby fostering healthy behavioral habits. Additionally, priority must be given to improving ventilation in communal areas such as classrooms and dormitories. Mechanical ventilation equipment should be installed in areas with poor airflow, and the management and maintenance of ultraviolet germicidal lamps must be standardized to ensure consistent efficacy. Through the systematic implementation of these multifaceted measures, the risk of TB transmission within schools can be significantly mitigated, ensuring a safe and healthy campus environment. At the same time, efforts should focus on disseminating knowledge about latent tuberculosis infection and preventive treatment, enhancing public understanding that “latent infection does not mean no risk.” Moreover, the effective implementation of latent infection control requires collaborative efforts among healthcare institutions, disease control authorities, schools, and families, supported by improved policy frameworks and strengthened doctor-patient communication, to increase both the acceptance and completion rates of preventive treatment.

## Data Availability

The datasets presented in this study can be found in online repositories. The names of the repository/repositories and accession number(s) are as follows: GSA (Genome Sequence Archive) at the National Genomics Data Center, China (https://ngdc.cncb.ac.cn/search/all?&q=CRA039100) accession number CRA039100.
