# Supplementary material for: A clustered pulmonary Tuberculosis outbreak at a technical school in Shenzhen, China
Source: Front Pediatr. 2026 May 7;14:1817016. doi: 10.3389/fped.2026.1817016 (PMC13190494; doi:10.3389/fped.2026.1817016)
Supplement: Supplementary file 1 [file Supplementaryfile1.docx]

**Supplementary materials**

**A Clustered Pulmonary Tuberculosis Outbreak in a Technical School in Shenzhen, China**

**Table S1 Demographic Characteristics of the Study Population (Stratified by Students/Staff)**

**Table S2 Completion and Results of the Initial Screening (Stratified by Students/Staff)**

**Table S3 Overall Screening Results (Stratified by Students/Staff)**

**Table S4. Distribution and comparison of screening results (IGRA and CXR) among student dormitories**

**Table S5 Results of Statistical Testing Against the Outbreak-Associated Dormitory**

**Table S1. Demographic Characteristics of the Study Population (Stratified by Students/Staff)**

| Variable | Students (N = 406) | Staff (N = 47) |
| --- | --- | --- |
| Sex | Male:234(57.6%); Female:172 (42.4%) | Male:22(46.8%); Female: 25 (53.2%) |
| Age (Years) | 16.7 ± 0.8 | 39.0 ± 12.3 |

**Table S2. Completion and Results of the Initial Screening (Stratified by Students/Staff)**

| Group | Total Target | IGRA Completed (%) | CXR Completed (%) |
| --- | --- | --- | --- |
| Students | 416 | 406 (97.6%) | 406 (97.6%) |
| Staff | 47 | 47 (100.0%) | 47 (100.0%) |
| Total | 463 | 453 (97.8%) | 453 (97.6%) |

CXR, Chest X-ray.

**Table S3. Overall Screening Results (Stratified by Students/Staff)**

| Group | IGRA Positive (%) | CXR Abnormal (%) |
| --- | --- | --- |
| Students | 40 (10.6%) | 12 (3.0%) |
| Staff | 8 (18.6%) | 1 (2.1%) |
| Total | 48 (11.4%) | 13 (2.9%) |

**Table S4. Distribution and comparison of screening results (IGRA and CXR) among student dormitories**

| **Dormitory** | **IGRA Positives / Tested (Positivity Rate)** | **CXR Abnormalities / Tested (Abnormality Rate)** |
| --- | --- | --- |
| 310^ | 5/5 (100.0%)^#^ | 3/5 (60.0%) |
| 309^ | 5/8 (62.5%) ^NS^ | 0/8 (0.0%) |
| 506^ | 3/6 (50.0%) ^NS^ | 1/6 (16.7%) |
| 308^ | 3/8 (37.5%) ^NS^ | 2/8 (25.0%) |
| 314 | 1/3 (33.3%) ^NS^ | 0/3 (0.0%) |
| 306 | 2/8 (25.0%) ^NS^ | 0/8 (0.0%) |
| 202 | 1/7 (14.3%) ^NS^ | 0/7 (0.0%) |
| 207 | 1/7 (14.3%) ^NS^ | 0/7 (0.0%) |
| 209 | 1/8 (12.5%) ^NS^ | 0/8 (0.0%) |
| 218 | 1/8 (12.5%) ^NS^ | 0/8 (0.0%) |
| 311 | 1/8 (12.5%) ^NS^ | 0/8 (0.0%) |
| 204 | 0/10 (0.0%)** | 0/10 (0.0%) |
| 307 | 0/8 (0.0%)** | 0/8 (0.0%) |

**Notes:** Significant markers: * P<0.05, * * P<0.01, * * P<0.001, NS showed no significant difference.^ Indicates dormitories occupied by students from Class 2302 (Juxian Building rooms 309, 506, 310, and 308).

**Table S5. Results of Statistical Testing Against the Outbreak-Associated Dormitory**

| Dormitory Comparison | OR | P value | P adjust |
| --- | --- | --- | --- |
| 309 VS 310 | Inf | 0.2308 | 1.00 |
| 506 VS 310 | 0.00 | 0.1818 | 1.00 |
| 308 VS 310 | Inf | 0.0754 | 0.9044 |
| 314 VS 310 | 0.00 | 0.1071 | 1.00 |
| 306 VS 310 | Inf | 0.0210 | 0.2517 |
| 202 VS 310 | Inf | 0.0152 | 0.1818 |
| 207 VS 310 | Inf | 0.0152 | 0.1818 |
| 209 VS 310 | Inf | 0.0047 | 0.0559 |
| 218 VS 310 | Inf | 0.0476 | 0.5714 |
| 311 VS 310 | 0.00 | 0.0047 | 0.0559 |
| 204 VS 310 | Inf | 0.0003 | 0.0040 |
| 307 VS 310 | Inf | 0.0008 | 0.0093 |
